# Reliable detection of low visual acuity in mice with pattern visually evoked potentials

**DOI:** 10.1038/s41598-018-34413-8

**Published:** 2018-10-29

**Authors:** Naoyuki Tokashiki, Koji M Nishiguchi, Kosuke Fujita, Kota Sato, Yurika Nakagawa, Toru Nakazawa

**Affiliations:** 1 Department of Ophthalmology, Tohoku University Graduate School of Medicine, Sendai, 980-8574 Japan; 2 Department of Advanced Ophthalmic Medicine, Tohoku University Graduate School of Medicine, Sendai, 980-8574 Japan; 3 Department of Retinal Disease Control, Tohoku University Graduate School of Medicine, Sendai, 980-8574 Japan; 4 Department of Ophthalmic Imaging and Information Analytics, Tohoku University Graduate School of Medicine, Sendai, 980-8574 Japan

## Abstract

Measuring the optokinetic response (OKR) to rotating sinusoidal gratings is becoming an increasingly common method to determine visual function thresholds in mice. This is possible also through direct electrophysiological recording of the response of the neurons in the visual cortex to the presentation of reversing patterned stimuli, i.e. the pattern visually evoked potential (pVEP). Herein, we optimized the conditions for recording pVEPs in wild-type mice: we investigated the optimal depth (1, 2, or 3 mm) of the inserted electrode and the optimal stimulus pattern (vertical, horizontal, or oblique black and white stripes, or a checkerboard pattern). Visual acuity was higher when measured with the optimal pVEP recording conditions, i.e., with the electrode at 2 mm and a vertical-stripe stimulus (0.530 ± 0.021 cycle/degree), than with OKR (0.455 ± 0.006 cycle/degree). Moreover, in murine eyes with optic nerve crush-induced low vision, OKR could not measure any visual acuity, while pVEPs allowed the reliable quantification of residual vision (0.064 ± 0.004 cycle/degree). Our results show that pVEPs allow more sensitive measurement of visual function than the OKR-based method. This technique should be particularly useful in mouse models of ocular disease and low vision.

## Introduction

A commercial semi-automated system for the measurement of visual acuity (Optomotry) was recently introduced and has quickly become commonly used for the behavioral evaluation of visual function in awake mice^1^. In this system, the optokinetic response (OKR) to rotating sinusoidal gratings is judged subjectively by a masked observer. The key features of the system include easy handling and simultaneous measurement of the right and left eyes in a relatively short time. This system has been used successfully to assess visual function in mice with retinal dysfunction^2–4^. OKR-measured visual acuity is dependent on the integrity of both the subcortical visual pathway and the visual cortex (V1), as bilateral ablation of the V1 only partially affects the visual outcome^5^. Therefore, it is not suitable for assessing visual acuity when cortical dysfunction is suspected. OKR measurements may also be affected by non-visual problems, including neural and muscular dysfunction related to movements of the eye or the neck. By contrast, measurement of the pattern visually evoked potential (pVEP) constitutes a direct measurement of the electrical response of the V1 to a patterned visual stimulus. The pVEP is reported to arise mostly from the activity of neurons in the V1^6^, but has also been used, less commonly compared to the OKR-based method, to quantify visual acuity^7–12^ Measuring the pVEP requires cranial surgery for electrode implantation and sedation of the mice during recording. It is not semi-automated, but the results are more objective and represent a direct assessment of the V1, thereby reflecting the integrity of the entire visual pathway, i.e., from the retina to the V1. To date, no study has compared measurements of visual acuity made with OKR and pVEPs.

This study set out, first, to optimize the recording conditions of pVEP measurement, and next, to compare measurements of visual acuity obtained with optimized pVEP conditions and with Optomotry. We found that pVEPs were more sensitive in measuring visual acuity and provided reliable results, particularly in animals with severe visual dysfunction.

## Results

**Figure 1:**
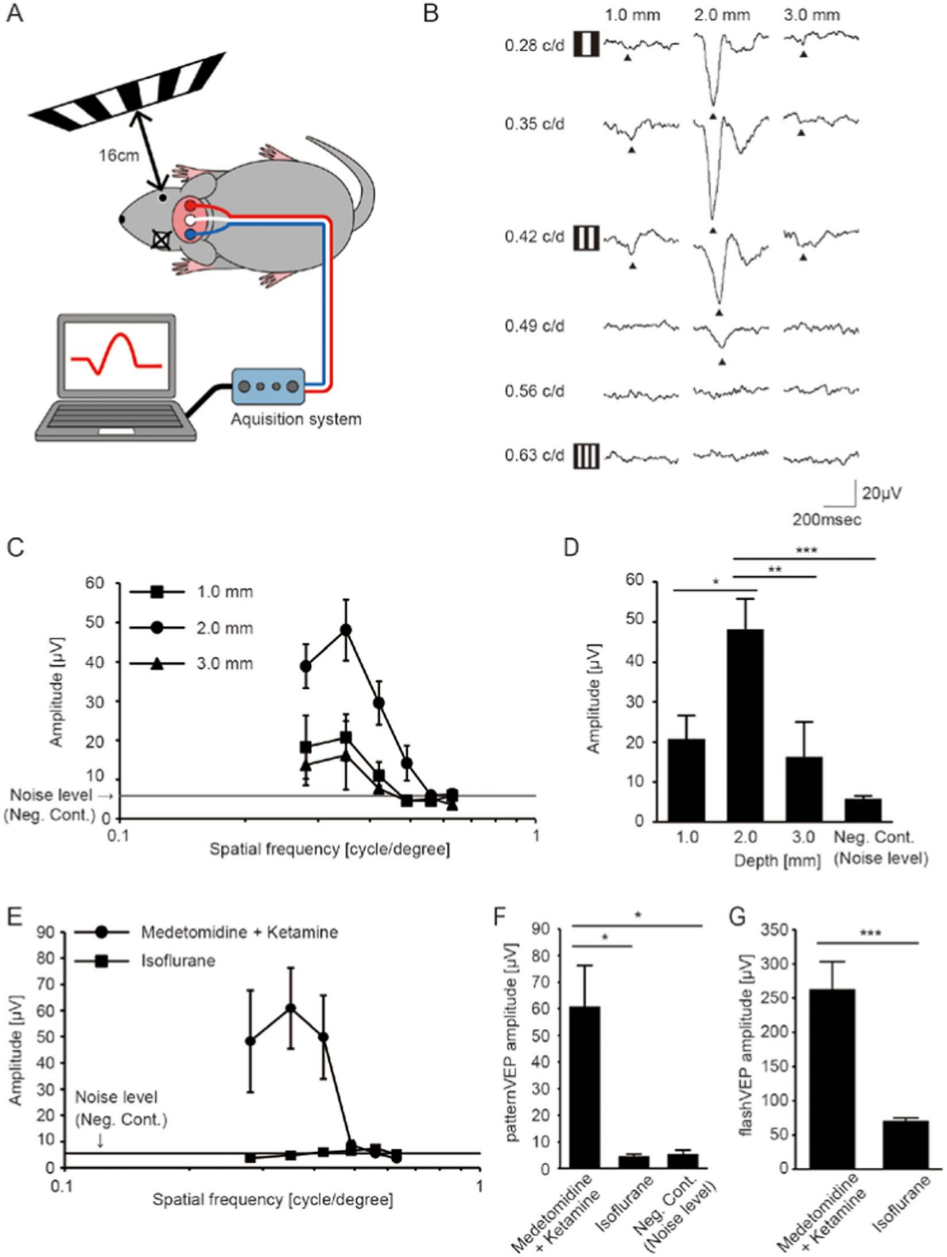
Optimization of pVEP recording conditions. (**A**) Image of experimental setup for measuring pVEPs. The monitor that showed the pattern stimulus was placed 16 cm away from the tested eye, perpendicular to the visual axis. The contralateral eye was completely sealed. (**B**) Representative pVEP traces recorded from electrodes inserted at three different depths. The 6 vertically aligned traces at each depth were recorded from the same mouse. The responses to the patterns of the lowest to the highest spatial frequencies are shown from the top to the bottom. The filled arrowheads indicate the first negative trough. (**C**) Quantification of pVEP amplitudes recorded from electrodes inserted at three different depths. Results for electrodes inserted at depths of 1.0 mm (square, N = 6), 2.0 mm (circle, N = 7) and 3.0 mm (triangle, N = 6) below the skull surface, into the V1, are presented. Note that the amplitude was largest at 0.35 cycle/degree. Noise level = 5.81 μV (N = 6). (**D**) Histogram of pVEP amplitudes in response to a 0.35 cycle/degree visual stimulus. The largest amplitude was recorded in the V1 with 2.0-mm deep electrodes. Negative controls (Neg. Cont.) signals were obtained from 12 mice. *P < 0.05, **P < 0.01, ***P < 0.001. ANOVA followed by Tukey-Kramer’s multiple comparison test. Data represent mean ± standard error of the mean. (**E**) Quantification of pVEP amplitudes recorded from mice using two different anesthetics: an intraparietal injection of medetomidine and ketamine (filled circle, N = 6) or an inhalation of isoflurane (filled square, N = 6). Noise level = 5.39 μV (N = 4). (**F**) Histogram of pVEP amplitudes in response to 0.35 cycle/degree visual stimulus. The same data were used for Neg. Cont. data and for designating the noise level (N = 4). *P < 0.05. ANOVA followed by Tukey-Kramer’s multiple comparison test was applied. Data represent mean ± S.E.M. (**G**) Histogram of flash VEP amplitudes in response to −1.0 log cd s/m^2^ flash stimulus. ***P < 0.001. The student’s t-test was applied. Data represent mean ± standard error of the mean.

**Figure 2:**
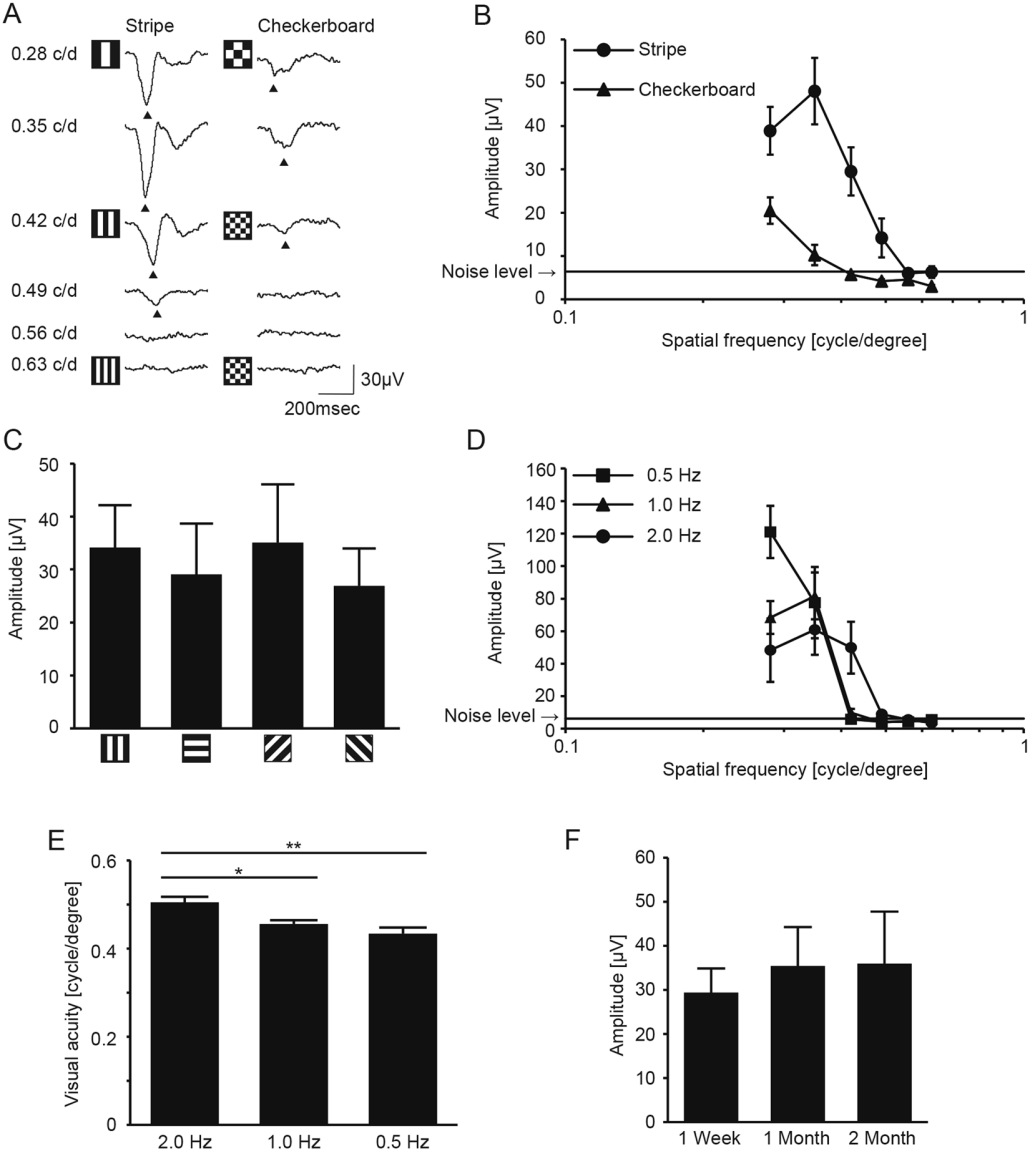
Comparison of stimulus patterns for measuring pVEPs. (**A**) Representative pVEP traces in response to vertical-stripe or checkerboard patterns. The spatial frequency of the stimulus (0.28–0.63 c/d) is shown on the left side. (**B**) Summary of pVEP amplitudes stimulated by vertical stripe or checkerboard patterns. The amplitude was largest at 0.35 cycle/degree for the vertical stripe pattern (filled circle, N = 7) and at 0.28 cycle/degree for the checkerboard pattern (filled triangle, N = 7). The amplitudes of both patterns decreased with increasing spatial frequency and eventually became indistinguishable from the noise level. The amplitudes for the checkerboard pattern were generally lower than those for the stripe pattern. Noise level = 6.38 μV (N = 6). (**C**) pVEP amplitudes stimulated by stripe patterns of different orientations. All the patterns were presented at a spatial resolution of 0.35 cycle/degree (N = 5 each). (**D**) pVEP recorded using visual stimuli presented at three different temporal frequencies. The amplitude decreased in response to higher spatial frequencies and eventually became indistinguishable from the noise level at 0.42 cycles/degree (0.5 Hz, square, N = 6 or 1.0 Hz, triangle, N = 6) or at 0.49 cycles/degree (2.0 Hz, circle, N = 6). Noise level = 6.20 μV (N = 9). (**E**) Effect of temporal frequency of the visual stimuli on the measured pVEP visual acuity. *P < 0.05, **P < 0.01. (**F**) pVEP amplitude measurement during the first 2 months after electrode insertion. Amplitudes at 1 week, 1 month, and 2 months following electrode insertion were comparable (*P* = 0.818). The numbers of mice assessed declined from 17 mice at 1 week to 14 mice at 1 month and 9 mice at 2 months due to the loss of the electrodes themselves, or access to them. Data represent mean ± standard error of the mean. ANOVA followed by Tukey-Kramer’s multiple comparison test was applied (**E**) and (**F**). Data represent mean ± standard error of the mean.

The set up used for recording pVEPs in the current study is presented in Fig. 1A. To date, only a small number of studies have examined visual acuity with the pVEP in mice^7–12^ and information related to the recording conditions is rather scarce. Thus, we first assessed various recording conditions for measuring pVEPs, including the depth of insertion of the electrode into the skull over the visual cortex using medetomidine and ketamine. We found that the recorded pVEP amplitudes were largest when the electrode was inserted to a depth of 2.0 mm below the skull surface compared to values obtained when the electrodes were inserted to depths of 1.0 mm and 3.0 mm (Fig. 1B–D). Therefore, the electrodes were implanted 2.0 mm into the skull in the following experiments. Furthermore, the pVEP was measured using isoflurane, an anesthetic for rodents widely used in neuroscience research (Fig. 1E), with the aim of expanding its applicable uses. However, pVEPs were virtually undetectable, showing no difference from noise (P = 0.999). We infer that this was due to the suppression of the cortical response^13^, because sequential recording of flash VEPs displayed a stronger signal than pVEPs; they were detectable but severely reduced (Fig. 1G). In humans, checkerboard-pattern visual stimuli are usually used to record pVEP^14^. This led us to compare pVEPs derived from vertical stripes and checkerboards in mice; we found that the pVEPs were much higher in the former than the latter (Fig. 2A,B). On the other hand, stimuli comprising stripes of various orientations resulted in pVEPs that did not differ (Fig. 2C). Next, we considered the temporal dynamics of the pVEPs. We assessed pVEPs evoked by visual stimuli at 0.5 Hz, 1.0 Hz, and 2.0 Hz, taking into account that waveform analysis of the peak and the trough requires a sweep of ~500 msec (2 Hz), and that corneal clouding can potentially have an adverse effect during extended recording time (Fig. 2D). Acuity measurements obtained in response to stimuli at 2.0 Hz (0.505 ± 0.030; mean ± standard deviation) were only slightly better than those in response to stimuli at 1.0 Hz (0.456 ± 0.022; mean ± standard deviation) and 0.5 Hz (0.434 ± 0.034; mean ± standard deviation, Fig. 2E). We also found that, once the electrodes were implanted, recording with stable results was possible for the next 2 months (Fig. 2F). However, the number of recordable electrodes was greatly reduced at 2 months, because they were either covered by regenerated skin or lost, and pVEPs were recordable in only 9 out of 17 mice (52.9%).

**Figure 3:**
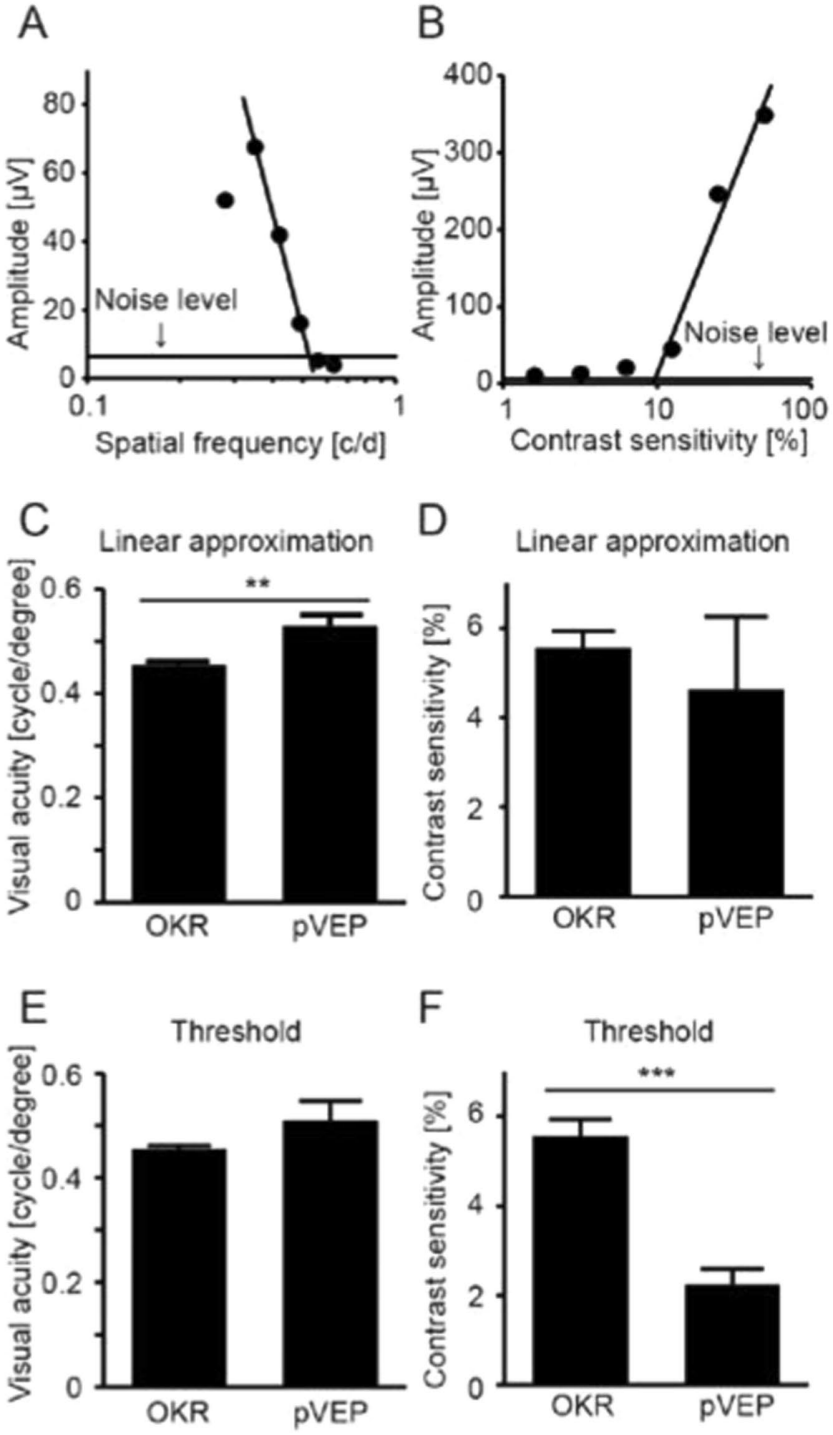
Comparison of visual acuity as measured with pVEPs and Optomotry. (**A**,**B**) Representative plots of pVEP amplitudes shown as a function of spatial frequency or contrast sensitivity of the stimulus pattern for a given mouse. Noise level = 6.38 μV for A (N = 7) and 5.99 μV for B (N = 5). (**C**,**D**) Comparison of visual function evaluated with pVEPs with linear approximation and Optomotry. Visual acuity was higher when measured with the former than the latter (*P* = 0.002). However, measurements of contrast sensitivity made with Optomotry and pVEPs were comparable (*P* = 0.504). (**E**,**F**) Comparison of threshold determined with pVEPs and OKR. The threshold of visual acuity was comparable when evaluated with pVEPs and OKR (P = 0.119). Conversely, the threshold of contrast sensitivity was lower when measured with pVEPs than with OKR (P = 0.0002). **P < 0.01, ***P < 0.001, Student’s *t*-test. N = 10 for Optomotry, N = 7 for visual acuity with pVEPs, and N = 5 for contrast sensitivity with pVEPs. Data represent mean ± standard error of the mean.

**Figure 4:**
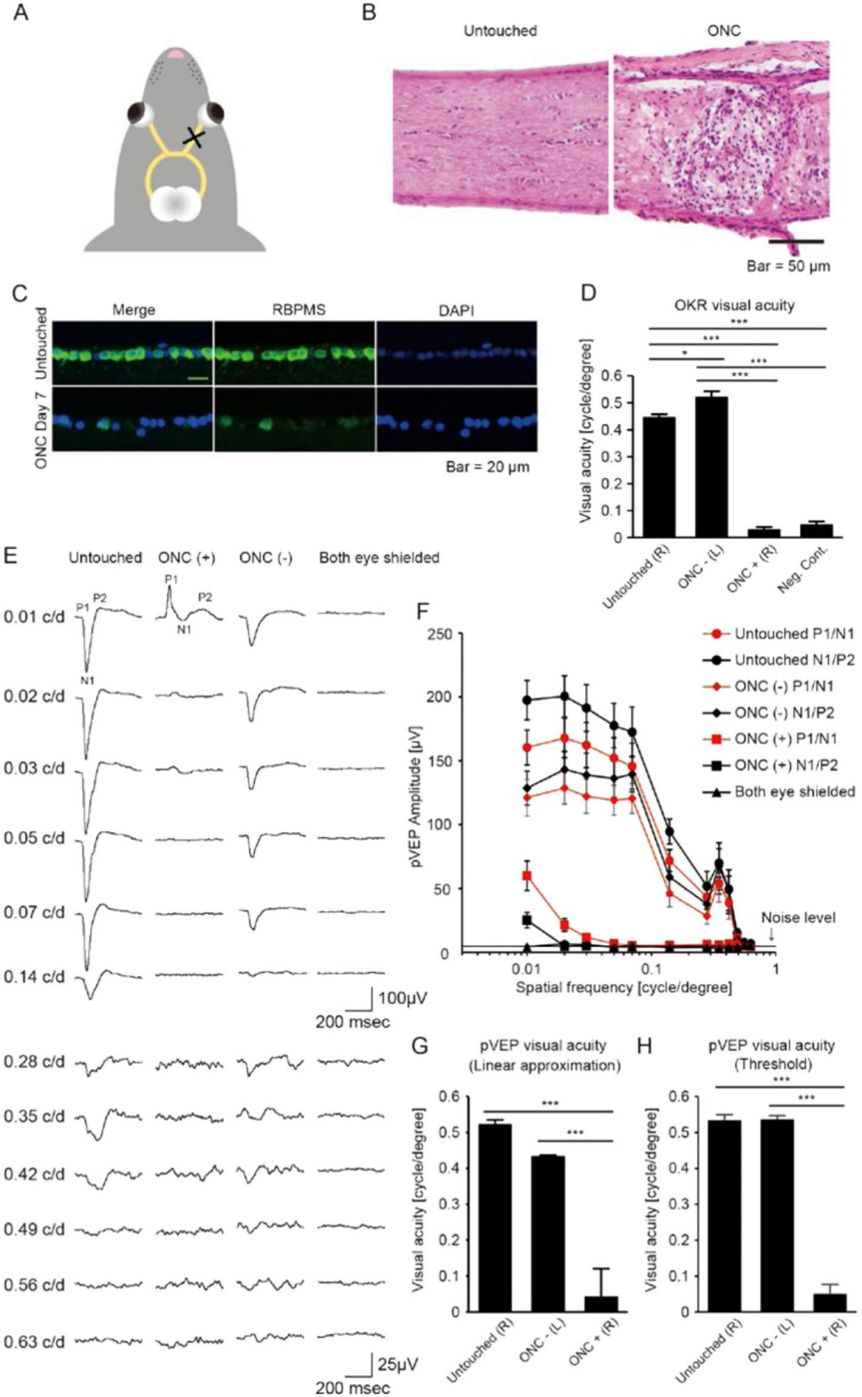
Reliable detection of residual visual acuity in mice with severe optic nerve damage. (**A**) Schematic illustration of optic nerve crush (ONC). The right optic nerve was pinched with tweezers behind the globe. (**B**) Histological sections with Hematoxylin-Eosin staining of the optic nerve, untouched and after ONC (right and left, respectively). Note that ONC destroyed the tissue and led to the infiltration of inflammatory cells. Scale bar: 50 μm. (**C**) Histological sections of the retinal ganglion cell (RGC) layer in an ONC eye. Rbpms-positive RGCs (green) were fewer 7 days after ONC. Scale bar: 20 μm. (**D**) OKR-measured visual acuity in the ONC model. Optomotry could not detect residual visual acuity after ONC (ONC + (R)). Note that measured visual acuity after ONC is comparable to the background noise level of the system (Neg. Cont.; *P* = 0.883. N = 10 for untouched mice (untouched (R)), N = 10 for ONC mice (ONC – (L) and ONC + (R)), and N = 5 for Neg. Cont. *P < 0.05, ***P < 0.001. (**E**) Representative pVEP traces recorded from the hemisphere contralateral to the ONC eye. pVEP from an untouched mouse and a blinded control (both eyes were shielded) are also shown. Note that the pVEP waveforms in the ONC mouse were very different from the untouched mouse. (**F**) Summary of pVEP amplitudes measured in ONC mice, shown as a function of the spatial frequency of the pattern stimulus. N = 9 for ONC mice, N = 7 for untouched mice, and N = 4 for mice with both eyes shielded. Noise level = 4.67 μV (N = 4). (**G**) Comparison of visual acuity in ONC and untouched eyes, as evaluated with linear regression of pVEP amplitudes. Note that visual acuity in the ONC eyes is lower than the untouched eyes. ***P < 0.01., N = 9 for ONC mice, N = 7 for untouched mice. Data represent mean ± standard error of the mean. Neg. Cont.: negative control. (**H**) Comparison of visual acuity in ONC and untouched eyes, as evaluated with threshold determination of pVEP responses. ***P < 0.001. ANOVA followed by Tukey-Kramer’s multiple comparison tests was applied for comparisons. Data represent mean ± standard error of the mean.

Having established the optimal pVEP recording conditions, we then measured visual acuity and contrast sensitivity, two fundamental indices of visual function, with pVEPs and Optomotry (a semi-automated system for measuring OKR-based visual function) in wild-type mice and compared the results (Fig. 3). We used two methods, a linear approximation (Fig. 3A,B) and threshold determination, to analyze the pVEP data in this test and calculate visual acuity and contrast sensitivity. Visual acuity as calculated with a linear approximation was slightly higher (Fig. 3C) when based on pVEP data (0.530 ± 0.021 cycles/degree) than on Optomotry data (0.455 ± 0.006 cycles/degree; *P* = 0.002). However, contrast sensitivity did not differ when measured with these two methods (Fig. 3D; *P* = 0.256). Results were slightly different when visual function was calculated with threshold determination. Visual acuity was similar when based on pVEP data (0.510 ± 0.037 cycles/degree) and Optomotry data (0.455 ± 0.006 cycles/degree; *P* = 0.119, Fig. 3E), whereas contrast sensitivity was slightly lower when based on pVEP data (2.240 ± 0.351%) than Optomotry data (5.556 ± 0.373%; *P* = 0.0002, Fig. 3F). Taken together, it thus appears that pVEPs may be a slightly more sensitive measure of visual function in mice. Next, we investigated whether pVEP measurement also allowed more sensitive detection than Optomotry of visual acuity in mice with low vision. To this end, we measured visual function in mice that underwent optic nerve crush (ONC), a model of low vision commonly used in retinal ganglion cell (RGC) research. In this model, the optic nerve is exposed and mechanically pinched by forceps behind the globe (Fig. 4A). This causes severe axonal damage (Fig. 4B) followed by the loss of RGCs (Fig. 4C). When we assessed visual function in post-ONC mice with Optomotry, we found that the measurements of visual acuity were indistinguishable from the noise level (Fig. 4D). On the contrary, clear pVEP signals were measurable (and similar) for both the trough (P1/N1) and the peak (N1/P2) even in response to stimuli of low spatial frequency, although the waveforms were severely altered (Fig. 4E,F). Moreover, these altered waveforms were clearly distinguishable from any possible contamination in the response caused by the contralateral healthy eye, which was shielded from the light. Thus, visual acuity was roughly similar when it was calculated with linear approximation and with threshold determination.

## Discussion

In this study, we found that visual acuity in mice may be higher when measured with pVEPs than with a widely used OKR-based behavioral test (Optomotry). The reasons for this may be, first, that the pVEP directly represents the neural function of the visual cortex, whereas OKR reflects the function of the subcortical visual pathway, not the visual cortex itself^5^. Importantly, OKR is affected also by the function of the efferent nervous system, which controls ocular and neck movements. Second, pVEP measurement is objective, whereas OKR is judged subjectively by an observer. Third, behavioral tests are often affected by non-visual cues, including the environment in which the recording takes place. However, the difference we observed may have arisen from technical differences in the two methods and not from differences in OKR behavior and cortical responses themselves, as we found that different methods of determining visual function, i.e., linear approximation and threshold determination, produced slightly different results.

An additional finding of this study was that pVEPs may be particularly useful for measuring visual acuity in mice with severe visual impairment, even when Optomotry fails altogether to reveal remaining vision. In mice that underwent ONC, we found that not only the response of the contralateral V1, measured by pVEP, was robust at low spatial resolutions, but also that its waveform was severely altered by the injury. This alteration allowed us to exclude any potential contaminating signal from the visual response of the healthy contralateral eye, which is possible since ~5% of retinal information is relayed ipsilaterally in mice^15^. Moreover, ONC affects all the retinal outputs, including those that project to the superior colliculus. As the superior colliculus is involved in movements of the eye and the head^15^, ONC might potentially impair the head tracking response to the rotating-grating stimulus, affecting the visual outcome. Thus, caution is required when interpreting visual acuity measurements made with OKR at least in this model, as they may not necessarily reflect only visual function.

This study included testing of various stimulus patterns for evoking a cortical response, with the aim of optimizing pVEP recording conditions. However, we found that the orientation of a stripe pattern resulted in no differences. In humans, a checkerboard pattern is often used to record pVEPs, as it yields the most reliable outcomes^14^. Contrary to our expectations, we found that pVEPs stimulated by a checkerboard pattern were weak in mice, although the reasons for this remain unclear.

In the past, another group measured OKR visual acuity in an ONC model and found virtually no measurable visual acuity^16^, which is consistent with our results. Visual acuity threshold measurement in a water maze has also been reported^17^, but this type of assay is labor-intensive^18^. Moreover, water mazes have been used only rarely in assessing visual acuity in disease models^19^ and there are no reports on their application in the ONC model. On the other hand, we have successfully detected visual acuity with the pVEP response to visual stimuli at low spatial frequencies in the ONC model. However, the waveforms we observed had grossly abnormal prominent positive peaks that were almost reciprocal to the prominent negative troughs in control mice. Although the origin of the pVEP waveform is not well understood, it is possible that alterations in the waveform reflect impairment of aspects of visual function other than spatial resolution, as tested in the current study.

The drawback of the pVEP system is that it requires some skill to insert electrodes into the brain of the animal, and to perform the electrophysiological recording. However, once the system is set up, it requires only 15 minutes for the implantation of the electrodes and a single recording session of 30 minutes to extract pVEP data from a single mouse. This compares favorably to Optomotry measurements, which, as used in this study, require four recording sessions of 20 minutes each, on four consecutive days. Another important drawback of pVEP is the additional discomfort it imposes on the mice compared to the Optomotry system. In this context, intrinsic signal optical imaging at the visual cortex that also allows assessment of detailed visual function, including visual acuity, may be less invasive to the animals^20–22^. However, considering the various advantages that pVEPs have over OKR-based measurements, we feel the technique deserves closer attention, and may be particularly suitable for measuring visual function in disease models of low vision, or those based on impairment of the optic nerve or the visual cortex.

In conclusion, this study found that measuring pVEPs was a sensitive and reliable method to assess visual acuity in mice, particularly in mice used as models of visual dysfunction.

## Methods

### Animals and mouse optic nerve crush (ONC) model

C57BL/6 J mice (male, 7–12 weeks old) were purchased from SLC Inc. (Hamamatsu, Japan). All animals were treated in accordance with the ARVO Statement guidelines for the Use of Animals in Ophthalmic and Vison Research, and the intramural Guidelines for Care and Use of Animals. Experimental procedures were conducted after approval by the Ethic Committee for Animal Experiments at Tohoku University Graduate School of Medicine. Cervical dislocation was applied to the sacrifice animals when necessary.

Before ONC, each mouse was anesthetized with an intraperitoneal injection of medetomidine (0.6 mg/kg; Meiji Seika Pharma Co. Ltd., Tokyo, Japan) and ketamine (36 mg/kg; Daiichi Sankyo Co. Ltd., Tokyo, Japan). Then, the eye was subluxated to expose the optic nerve, which was pinched with tweezers 2 mm behind the globe for 5 seconds, as previously described^2,23^. Finally, the eye was returned to the original position and a fundus examination was performed to rule out potential retinal ischemia induced by the procedure.

### Assessment of visual function with pVEPs and Optomotry

pVEPs were recorded with a similar system as that used for measuring flash VEPs, as previously described in greater detail, with modifications^24^. After each mouse was anesthetized as above, the skin was removed from the top of the head to expose the skull. Two microholes were opened, 3.6 mm caudal to and 2.3 mm lateral from, the bregma with a drill (M 0.5 mm)^1^. Two stainless steel pen-head screws (M 0.6 mm) were inserted into these holes, to depths of 1.0 mm, 2.0 mm, or 3.0 mm. These screws were fixed in place using cyanoacrylate adhesive (Toagosei Co. Ltd, Tokyo, Japan). Dental cement (GC Unifast III; GC Dental Products Corp., Tokyo, Japan) was applied to the exposed skull to seal the wound. One week after the electrode implantation, pVEPs were recorded using an acquisition system (PuREC; Mayo Corp., Inazawa, Japan) and a display toolbox (VPixx; VPixx technologies, Saint-Bruno, QC Canada). After the mice were dark-adapted overnight, the pupil of the tested eye was dilated with 0.4% tropicamide eye drops (Santen Pharma Co. Ltd., Osaka, Japan) followed by the instillation of moisturizing eye drops (3% sodium hyaluronate and 4% sodium chondroitin sulfate; Alcon Laboratories, Fort Worth, TX). Then, the mice were anesthetized with an intraperitoneal injection of medetomidine and ketamine, as described above, or with an inhalation of isoflurane (1 ml/1 ml; Pfizer Co. Ltd., Tokyo, Japan). pVEPs were recorded one eye at a time. The electrode implanted into the visual cortex contralateral to the tested eye served as the positive electrode and the ipsilateral electrode was used as the negative to minimize background noise. A ground electrode, on the tail, was also connected to the acquisition system. pVEPs were recorded by placing a 19-inch monitor (S1933; Eizo, Hakusan, Japan) parallel to and 16 cm away from the plane of the stimulated eye. The contralateral eye was completely covered to assure that no light entered this eye. The monitor displayed square-wave gratings with an intensity of 3.0 cd/m^2^ for the dark bars and 159 cd/m^2^ for the light bars. Contrast was kept constant at 100% and the spatial frequency was varied, set at either 0.01, 0.02, 0.03, 0.05, 0.07, 0.14, 0.21, 0.28, 0.35, 0.42, 0.49, 0.56, or 0.63 cycles/degree of visual angle. Separately, spatial frequency was kept constant at 0.128 cycle/degree and contrast was varied, set at either 50, 25, 12.5, 6.3, 3.1 or 1.5%. The visual stimulus pattern gamma corrected for the monitor was reversed at a frequency of 2 Hz, unless indicated otherwise. The recorded electrical responses were band-pass filtered at 0.3 and 50 Hz, and a 60-Hz notch filter was applied. The average of 400 measured responses to each stimulus was used in the analysis. Moisturizing eye drops were washed out using physiological saline before recording the pVEPs, and saline was instilled to keep the tested eye moisturized during each interval. The amplitudes for the positive peak (N1-P2) were plotted (vertically) as a function of the spatial resolution or contrast sensitivity of the stimulus (horizontally).

Visual acuity or contrast sensitivity were determined primarily by drawing a linear regression line using 3 successive measurements, including the measurement with the maximum pVEP amplitude and the neighboring measurements for either higher spatial frequency or lower contrast, respectively, and identifying their intersection with the background noise. Visual acuity was also ascertained by determining the threshold of the response to the visual stimulus with the highest spatial frequency, selected from recognizable light responses.

fVEPs were recorded with the same acquisition system as above (PuREC; Mayo Corp.), with a Ganzfeld dome and a stimulator (LS-100) that showed white flashes at −1.0 log cd s/m^2^, presented at 0.016 Hz. Three sweeps were averaged to yield fVEP.

The noise level was determined in each independent experiment according to the largest positive peak amplitude observed in the mice when both eyes were shielded and the monitor was completely covered by a black curtain. These measurements of amplitude were averaged to calculate the noise level for each experiment.

Visual function was also measured with Optomotry (Cerebral Mechanics Inc., Canada) by observing the OKR of the mice to a rotating sinusoidal grating displayed on four monitors (average luminance: 81.0 cd/m^2^) surrounding the animal, as previously described^4^. The sinusoidal vertical grating had various spatial resolutions and contrast ratios, to determine the functional threshold of vision. In general, the sinusoidal vertical gratings had higher spatial frequencies than the square-wave gratings used to measure pVEPs. Four sessions were carried out on four consecutive days and the results averaged to measure visual acuity and contrast sensitivity. This test yielded independent measures of visual function for the right and left eyes, based on the differing sensitivity of each eye to the direction of pattern rotation (the stronger sensitivity to rotation in the temporal-to-nasal direction dominates the tracking response)^25^. Negative control data for OKR were obtained by running the program without a mouse present. The observer randomly selected the OKR direction, let the program run until it stopped, and assigned “visual acuity”.

### Histology

The eyes were processed for histological evaluation as previously described^26^. In brief, the removed eyes were put into tubes with a polyvinyl alcohol and polyethylene glycol compound and frozen in liquid nitrogen. Cryosections were stained with Hematoxylin-Eosin or subjected to immunohistochemical analysis using an anti-RBPMS antibody (#ab194213, 1:100; Abcam, Cambridge, UK) as the primary antibody. Donkey anti-rabbit-Alexa 488 (A21206, 1:500; Molecular Probes, Eugene, Oregon, USA) was used as the secondary antibody. The sections were mounted on a Vectashield mounting medium containing DAPI (Vector Laboratories, Burlingame, CA, USA).

### Statistical analysis

Statistical analysis was performed with JMP Pro 13 software (SAS Institute Inc.) for Windows. Statistical comparisons were made with the Student’s *t*-test or with a one-way analysis of variance followed by Tukey-Kramer’s multiple comparison test. The significance levels were set at P < 0.05 (*), 0.01 (**), and 0.001 (***).
